# Reassessing the Aging Enterprise: New Perspectives on the Social Construction of Later Life

**DOI:** 10.1093/geroni/igaa057.2539

**Published:** 2020-12-16

**Authors:** Chris Phillipson

**Affiliations:** Manchester Institute for Collaborative Research into Ageing, Manchester, England, United Kingdom

## Abstract

Four decades on from the publication of 'The Ageing Enterprise', this paper provides a critical review of the relationship between social theory and social policies for later life. To what extent do current theoretical perspectives in gerontology bear the influence of ideas laid out in that pioneering book? How has the ‘ageing enterprise’ fared given the dominant ideology of neo-liberalism and the precarious lives faced by people moving through the life course? The paper considers these questions in the context of globalization processes, and the imposition of austerity policies. The paper will consider the continuing importance of ‘The Ageing Enterprise’ by reviewing three main themes: first, assessing the changing relationship between the state and social policy; second, through examining current perspectives within critical gerontology; third, highlighting new forms of empowerment developing amongst older people, and the relationship of these to the values and ideas expressed in ‘The Ageing Enterprise’. Part of a symposium sponsored by the Women's Issues Interest Group.

